# Comment on “Fibroblasts in Nodular Sclerosing Classical Hodgkin Lymphoma Are Defined by a Specific Phenotype and Protect Tumor Cells from Brentuximab-Vedotin Induced Injury” *Cancers* 2019, *11*, 1687

**DOI:** 10.3390/cancers12020343

**Published:** 2020-02-03

**Authors:** Donatella Aldinucci, Alfonso Colombatti

**Affiliations:** Molecular Oncology Unit, Centro di Riferimento Oncologico di Aviano (CRO) IRCCS, PN, I-33081 Aviano, Italy; acolombatti@cro.it

We read with great interest the recent publication by Bankov et al. where they described the characteristics and functional activity of fibroblasts in Nodular Sclerosis (NS) Classical Hodgkin Lymphoma (cHL) [1]. The authors compared the gene expression and methylation profiles of fibroblasts isolated from primary lymph node suspensions and from lymphadenitis, founding significant differences, including a down regulation of the IL-7R gene and a strong up regulation of tissue inhibitor of metalloproteinase 3 (TIMP3). The authors reported that conditioned medium from Hodgkin and Reed Sternberg (HRS) tumor cells increased NS cHL fibroblast proliferation and that tumor cells were protected against the cytotoxic effects of Brentuximab-Vedotin by cHL fibroblasts [1]. 

We appreciate that the study of Bankov et al. [1] confirmed the protective effect of cHL fibroblasts against anticancer drugs [1,2] and the capability of conditioned medium from HRS cells to stimulate the growth of stromal cells derived from cHL lymph nodes [1,3].

However the authors asserted in the Results section that “IL-7R was 2.7-fold down regulated in NS cHL fibroblasts, which was previously described to be unregulated in NS cHL fibroblasts in one publication by Cattaruzza et al. [15]”, but in our manuscript published in *Int. J. Cancer* [4] in 2009 we only reported that IL-7 R is expressed by cHL fibroblasts [4]. Moreover, the authors asserted in the Discussion section that “It has been described that IL-7 secreted from HRS cells strongly induced proliferation of fibroblasts [15]”; however, we could not confirm this observation when adding purified IL-7 to the culture. In our manuscript [4] we reported that, in agreement with Iwata et al. [5], IL-6 secretion by cHL-fibroblasts was increased by IL-7 and by the co-cultivation of L-1236 cells (IL-7 secreting HRS cells) with cHL-fibroblasts [4].

In conclusion, we consider that there was a misinterpretation of our data since we have never claimed that IL-7R is up regulated in cHL fibroblasts or demonstrated that IL-7 secreted by HRS cells strongly induced the proliferation of cHL fibroblasts [4].
